# Ultra-Fine Scale Spatially-Integrated Mapping of Habitat and Occupancy Using Structure-From-Motion

**DOI:** 10.1371/journal.pone.0166773

**Published:** 2017-01-11

**Authors:** Philip McDowall, Heather J. Lynch

**Affiliations:** Department of Ecology and Evolution, Stony Brook University, Stony Brook, New York, United States of America; University of Sydney, AUSTRALIA

## Abstract

Organisms respond to and often simultaneously modify their environment. While these interactions are apparent at the landscape extent, the driving mechanisms often occur at very fine spatial scales. Structure-from-Motion (SfM), a computer vision technique, allows the simultaneous mapping of organisms and fine scale habitat, and will greatly improve our understanding of habitat suitability, ecophysiology, and the bi-directional relationship between geomorphology and habitat use. SfM can be used to create high-resolution (centimeter-scale) three-dimensional (3D) habitat models at low cost. These models can capture the abiotic conditions formed by terrain and simultaneously record the position of individual organisms within that terrain. While coloniality is common in seabird species, we have a poor understanding of the extent to which dense breeding aggregations are driven by fine-scale active aggregation or limited suitable habitat. We demonstrate the use of SfM for fine-scale habitat suitability by reconstructing the locations of nests in a gentoo penguin colony and fitting models that explicitly account for conspecific attraction. The resulting digital elevation models (DEMs) are used as covariates in an inhomogeneous hybrid point process model. We find that gentoo penguin nest site selection is a function of the topography of the landscape, but that nests are far more aggregated than would be expected based on terrain alone, suggesting a strong role of behavioral aggregation in driving coloniality in this species. This integrated mapping of organisms and fine scale habitat will greatly improve our understanding of fine-scale habitat suitability, ecophysiology, and the complex bi-directional relationship between geomorphology and habitat use.

## Introduction

Habitat suitability models for plants and animals often focus on course-grained abiotic habitat characteristics at the expense of microhabitat factors and/or biotic interactions that can also be important for structuring the use of space [1,2]. Despite their ubiquity and importance for spatial ecology, the scale and extent of data used to explore relationships between organisms and the space they occupy are often dictated by the availability of environmental data rather than the ecology and physiology of the organism under consideration [3]. Moreover, since data on environmental conditions and the presence/absence of the organism are usually recorded independently, there can be considerable spatial and temporal alignment errors between data types, making it difficult to infer the true relationship between them [1,4]. Issues of scale or spatiotemporal registration errors are relatively minor when key environmental covariates vary slowly (e.g., elevation) or when the spatial scale of occupancy is large (e.g., an island), but they can be highly problematic when modeling habitat suitability or space use at much smaller spatial scales, where occupancy may hinge on the detailed hydrology of the site or subtle variations in exposure to wind scour or solar irradiation [5].

Microclimate is likely to be critical in driving occupancy and abundance of a range of plants and animals because the environment as experienced by an individual can be very different from the average background condition measured at much larger spatial scales [6]. When high-resolution topographic maps are combined with climatic modeling, we may begin to detect fine scale variations that drive habitat associations. Seabra *et al*. [7] demonstrated that limpets at equal tidal heights and separated by less than two meters may experience significantly different incident solar radiation depending on which side of a rock they inhabit. The micro-scale differences in resulting temperature, which can be critical for the organisms as they perceive their environment, are far smaller than could be mapped using traditional air or water temperature datasets derived from satellite-based sensors. Terrestrial ecology also provides many such examples; different species of Anoline lizard occupy distinct microhabitats based on shade availability [8], the distribution of saxicolous lichens in the Rocky Mountains appear to be driven by fine scale variability in snow cover across the faces of boulders [9], and thermally constrained butterflies select topographically-driven microhabitat based on fine scale variations in temperature [10]. Topography can shape microclimate and provide fine-scale habitat that falls within the physiological tolerances of a species in an otherwise apparently unsuitable landscape. While these microhabitat drivers may not be as valuable as regional scale variables in predicting occupancy over large geographic ranges, they may be key to understanding small-scale interactions that structure the use of space by animals, and may improve model performance when used in conjunction with these regional variables [11,12]

Biotic interactions can also influence how animals and plants use space, making it difficult to infer the strength of abiotic habitat associations. Positive associations between conspecifics, either caused by active behavioral aggregation such as colonial breeding or the more passive dispersal limitations often seen in plant distributions, should lead to an increased density of individuals. Alternatively, negative associations, such as competition for resources, territorial behavior, or allelopathy should result in lower densities of individuals that are more regularly spaced across the landscape. When these biotic interactions are excluded from habitat suitability models, we risk erroneously assigning this variance to some landscape factor, and may find that our models perform poorly at predicting the spatial distribution of a species [2]. Similarly, if we do not explicitly include abiotic landscape heterogeneity into models of aggregation then we may incorrectly attribute patterns of aggregation to complex biotic interactions.

The use of topography as an explanatory variable in distribution modeling has a long history in the field of gradient analysis, whereby the abundance of a plant species is related to environmental gradients such as elevation. While most gradient analysis studies focus on the landscape scale, important fine scale details can be lost when data are collected at such large scales [13] as small scale heterogeneity in the landscape has been shown to promote species richness and beta diversity in plant communities [14]. In addition to information lost due to inappropriate scale, ecological datasets almost always project a three-dimensional landscape onto a two-dimensional raster, preventing a complete consideration of covariates that require a three-dimensional understanding of habitat (e.g., terrain, canopy structure, etc.) [14]. The additional information provided by digital elevation models (DEMs) can often be used as proxies for environmental condition and permit direct modeling of many processes, such as hydrological flow, critical to a site's suitability. To fully understand the spatial ecology of a species, it is important to map both organisms and the fine scale three-dimensional details of their landscape simultaneously. These maps, which we refer to as ‘integrated’ terrain and occupancy maps, are likely to be useful across a range of applications including ecology, ecophysiology, and biogeomorphology.

### Mapping fine-scale terrain

One of the basic structuring elements of habitat is elevation, however the resolution of available elevation data is highly variable across the globe. Within the United States, the USGS National Elevation Dataset provides low-resolution elevation data with relatively high spatial coverage, and a smaller number of high-resolution data products derived from aerial photogrammetry (with horizontal resolution up to 3 m). While similar data sets are available from several national agencies and commercial providers, global coverage is limited and high quality data are often hard to find and/or may be prohibitively expensive. Many of the more readily available products are derived from sources such as RadarSat and yield elevation datasets with horizontal resolutions in the range of hundreds or even thousands of meters. At these scales it is unlikely that recorded environmental variables accurately reflect the environment as experienced by an organism, as variation in microhabitat can lead to very different conditions existing at the smallest scales. Stereo pairs of imagery from commercial satellite images can be used to construct digital elevation models with spatial resolutions on the order of meters, but imagery at this resolution can be prohibitively expensive and cloud cover and shadows can create holes in the imagery that must be imputed.

LiDAR technology is capable of recording high-resolution 3D landscape structure [15] and it has been shown that such high resolution information on vertical structure can improve habitat suitability modeling studies [12,16,17], but LiDAR surveys entail equipment costs and often flight times that can be economically or logistically prohibitive. Structure-from-Motion (SfM), a computer vision technique, can be used to rapidly and economically produce detailed 3D information on the structure of the landscape [18] and simultaneously record the location of organisms within that landscape. SfM is simple enough to deploy in any landscape [19], and provides data on fine scale habitat characteristics that are otherwise unavailable. While this survey method has been used to create digital models of artifacts in archeology [20], measure canopy cover [21,22,23] and record coral morphology [19] in ecology, this work represents the first time the lower cost methodology of SfM has been used for modeling habitat suitability.

### Modeling conspecific interactions

While integrated terrain and occupancy models would find utility across a number of fields in ecology, we demonstrate its use by applying it to the study of conspecific attraction in colonial seabirds. Occupancy can be modeled either as a binomial process on a two-dimensional grid or as an inhomogeneous spatial point process; both approaches are made feasible using SfM, however we use a point process approach to demonstrate the use of SfM in a case study of nest site selection in the gentoo penguin (*Pygoscelis papua*). As in many colonially nesting birds, it is difficult to determine to what extent gentoo penguin nests are clumped due to active behavioral aggregation or whether they are simply responding independently to patchiness in suitable nesting habitat. High resolution terrain models integrated with information on patch occupancy allow us to quantitatively estimate the strength of these two competing hypotheses and provide an ecologically important case study for the use of this integrated mapping technique.

## Methods

Permits for this work were obtained from the National Science Foundation Division of Polar Programs in accordance with the Antarctic Conservation Act and Antarctic Treaty System.

### Structure-from-motion

**Fig 1:**
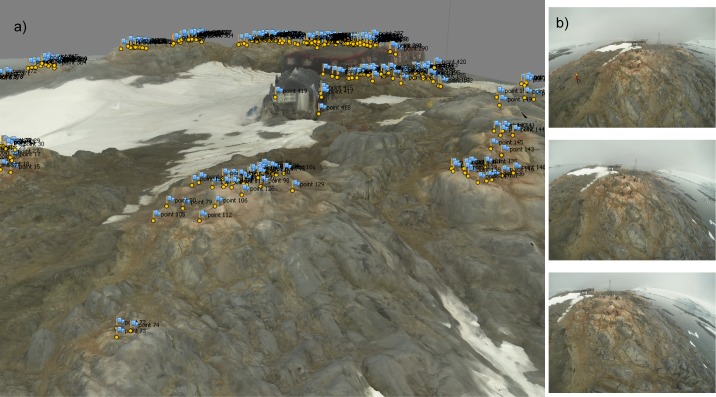
(a) Textured 3D mesh of Port Lockroy, Antarctica created from 493 images (b) Sample of images captured using a GoPro 3. Locations of occupied penguin nests are marked with yellow dots and blue flags.

Structure-from-Motion, a computer vision technique in which 3D structure is estimated from a set of overlapping images of the landscape, was used to produce a high-resolution 3D model of the study site with embedded information on nest locations (Fig 1). No prior information on the position from which images are captured is required, as the SfM algorithm is able to estimate the position of the cameras independent of the unknown 3D scene and, consequently, the structure of the 3D terrain.

We use a commercial product, Photoscan Professional Edition [24], for the photogrammetric workflow. Educational licensing is available for this product at a reduced rate, and in 2016 was priced at $59 for the standard edition and $549 for the professional edition. Alternative freely available software include visualsfm [25,26], tools in the python module OpenCV, and a selection of free-to-use web based services such as AutoDesk Catch 123D. All of these options use similar processes and workflows, but have different strengths and weaknesses [27]. Having tested many of these options, we think Photoscan provides the most complete and user friendly set of features for working with spatial data and producing georeferenced output from 3D reconstruction.

**Fig 2:**
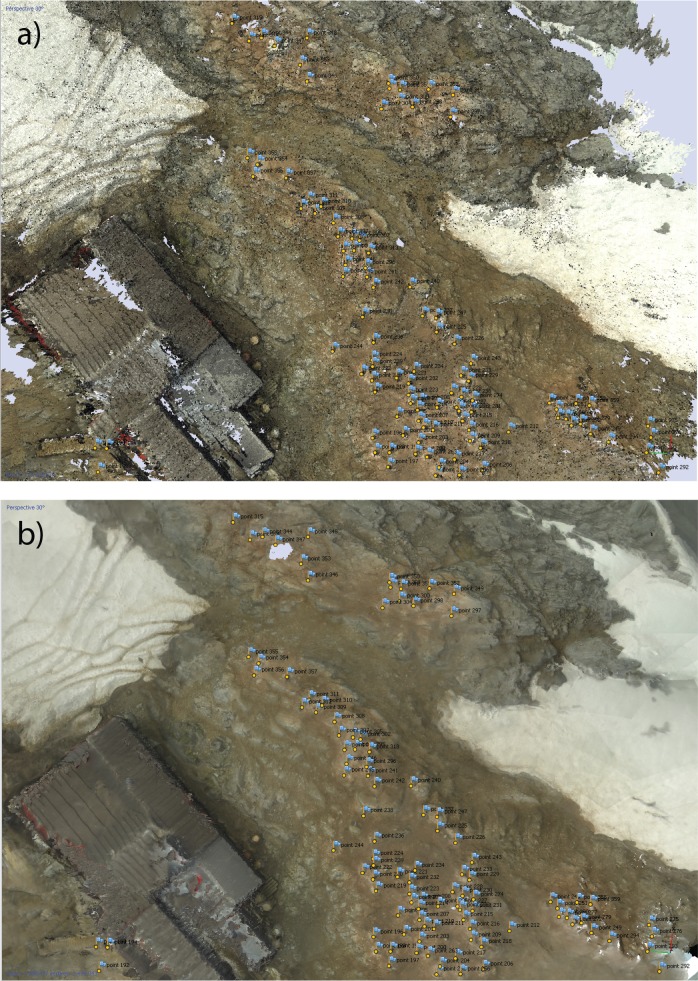
(a) Dense point cloud of Port Lockroy containing 113,338,579 points produced using 493 images processed with Photoscan (Agisoft). (b) Textured mesh containg 2,495,043 vertices fitted to dense point cloud.

Feature matching algorithms such as Scale Invariant Feature Transformations [28] are used to automatically identify corresponding points observed in multiple images and solve for the position of pairs of camera locations independently of the unknown structure of the 3D scene. Given the positions and orientation of the cameras from which the images were taken the software can project each matched point back into 3D space to yield a vector along which this point must lie. For each matched point we expect the intersection of the projections from multiple images to converge, giving the location of that point in 3D space. The estimated camera positions are then passed to a multi-view stereo algorithm to create a dense point cloud representing the 3D surface (Fig 2A). This dense point cloud is used as the basis for the generation of a polygon mesh, a 3D surface consisting of polygons that interpolates between the points in the dense point cloud (Fig 2). Though the mesh is in an arbitrary coordinate system, this mesh can be transformed into a real-world coordinate system through the use of additional information collected during the survey and an appropriate georectification method. When using cameras with a built-in GPS system, the metadata attached to each image can be used to estimate the position of the cameras. These estimated locations can then be used to transform the mesh to a real world coordinate system, while providing estimates of errors in camera locations. It should be noted, however, that these estimated errors convolve errors in the GPS positions and in the reconstructed mesh. Alternatively, information on the real-world position of points within the reconstructed scene can be used to georectify the model. Coded machine-readable targets, which can be produced automatically by Photoscan, can be placed into the scene prior to surveying and their positions recorded via a GPS (or differential GPS) unit. These targets can be automatically detected by the Photoscan software and encode an identifying number allowing unique markers to be identified. The model can then be georectified to these known points. These markers, when visible in multiple overlapping images, can also aid in the alignment of images and estimation of camera positions. If coded targets are not available, any recognizable point within the scene, or identifiable non-coded markers, can be used, although this requires the user to manually identify the targets within images and set their real-world coordinates. Once a 3D mesh has been produced and georectified, it can be converted into a raster DEM for convenient use in GIS software.

If no positional information is available at the time of surveying it is possible to export the model in an arbitrary coordinate system to GIS software where it can be georectified to satellite imagery as long as corresponding features, such as the corners of buildings, can be identified in both the 3D model and the satellite imagery. If this is the case, as it was in this study, it is important that the data are rescaled in the z-axis as georectification outside the SfM software will leave the elevation values in the original unscaled coordinate system. We exported an orthorectified image to ArcGIS and georectified it to existing 0.5 m resolution panchromatic satellite images using a linear transformation.

The ideal approach to SfM is to produce images using an aerial survey either from aircraft or unmanned aerial systems (UASs). However, due to cost considerations, frequent high winds, and risk of negative interactions between wildlife and UASs, we use a GoPro Hero 3 mounted on a 2.7 m pole that is carried around the site, with the camera capturing images of the terrain every ten seconds. While these oblique images are not ideal for SfM, tests showed that the elevation provided by the pole system was sufficient to allow reconstruction of the terrain. In an ideal scenario, a systematic transect survey would be planned in advance to ensure complete coverage; however, the opportunistic nature of field site access in the Antarctic and the need to avoid disturbing potentially sensitive nesting birds made this impossible for our application. To compensate for the lack of a systematic survey plan we collected many more images than necessary to ensure that we had complete coverage and sufficient views of all terrain. Despite the challenges of three-dimensional mapping across such complex terrain we managed to reconstruct an area of over 50,000 m^2^ in with only around 40 minutes of survey effort.

### Occupancy

After estimating the camera locations, we can project any given point in an image back into 3D space, resulting in a vector along which that point must lie. If we calculate the 3D coordinate of the intersection of this vector and the 3D mesh, we can place the point into the 3D landscape.

We selected a subset of the images that contained multiple projections of every occupied nest at the study site. The viewpoint afforded by the 2.7 m pole was sufficient to ensure that there was no occlusion of nests, and while we cannot always distinguish between a resting non-breeder (juvenile) and a nest with eggs being incubated by a breeding penguin, the use of expert interpretation of images minimizes false positives. In each of the images the pixel coordinates of each nest were used to project back to an intersection point on the 3D mesh. This step is possible directly in the Photoscan software package through marker placement on an image after generation of the mesh. This approach results in a set of points on the 3D surface corresponding to the estimated locations of all penguin nests in the site. Due to errors in alignment and the fact that different points within each nest are selected and projected onto the 3D mesh from different viewpoints, nests are occasionally recorded as a cluster of points. We filter these points assuming a minimum distance (10 cm) between the centers of adjacent nests, visually check the images to ensure each cluster is in fact an artifact of using multiple projections for a single nest, and then reduce these clusters to a single point at the center of the cluster. We can also use the total number of nests counted on the ground during surveying as a check on the number of clusters identified. The latitude and longitude of each point is then exported to GIS software.

### Point process modeling

Ripley’s K-function [29], K(r), a measure of the average number of points occurring within a radius ‘r’ of any other point, was used to provide a visual representation of pattern present. The value of Ripley’s K can be assessed over a range of values of ‘r’ and compared against a theoretical value expected under complete spatial randomness to understand scale dependent patterns occurring within the point pattern.

Mapping individual penguin nest locations also allows us to model the location of nests as the outcome of a spatial point process. We hypothesize that the observed clumping of *Pygoscelis* penguin individuals (both at the scale of the colony and sub-colony units) is a convolution of preference for auto-correlated terrain (specifically, well-draining areas at the top of local peaks in elevation) and significant levels of conspecific attraction. We model nest locations as the outcome of a hybrid Gibbs point process [30]. This model allows for interactions among points even as the intensity of the point process varies according to the underlying abiotic landscape features mapped using SfM. The hybrid model has three components; hard-core repulsion that prevents points from occurring within a distance *h* of each other, a Strauss interaction in which points separated by a distance between *h* and a radius *r*_1_ contribute a factor *γ*_1_ (*γ*_1_ < 1), to the probability density, resulting in a decreased probability of inter-point distances being found within this range, and a Strauss interaction in which points separated by a distance between *r*_1_ and a radius *r*_2_ contribute a factor *γ*_2_ (*γ*_2_ > 1), resulting in an increased probability of points within this distance. The probability density for the hybrid process is:

$$f(Ζ)=\alpha \left[\prod_{i=1}^{n}\beta ({Ζ}_{i})\right]\left[\prod_{i<j}\psi ({Ζ}_{i},{Ζ}_{j})\right]$$

where Z = (Z_1_,…,Z_*n*_) is the set of *n* points in the observed point process, *α* is a normalizing constant, and *ψ*(Z_*i*_,Z_*j*_) represents the pairwise interaction between points that depends on the distance ‖Z_*i*_ − Z_*j*_‖ between the points

$$\psi ({Ζ}_{i},{Ζ}_{j})=\left\{ \begin{array}{c} 0,\;\|{Ζ}_{i}-{Ζ}_{j}\|\leq h\\ \gamma_{1},h<\|{Ζ}_{i}-{Ζ}_{j}\|\leq r_{1}\\ \gamma_{2},r_{1}<\|{Ζ}_{i}-{Ζ}_{j}\|\leq r_{2}\\ 1,r_{2}<\|{Ζ}_{i}-{Ζ}_{j}\| \end{array}\right.$$

with *γ* > 1 representing attraction. The function *β*(Z_*i*_) is related to the first order intensity of the point pattern at the point locations Z_*i*_,

$$log(\beta ({Ζ}_{i}))=\mu +\sum_{k=1}^{l}\rho_{k}x_{k,i},$$

where *μ* is an intercept term, *ρ*_*k*_ are the coefficients for the set of *l* environmental covariates, and *x*_*k*,*i*_ are the values of the *k*^*th*^ environmental covariate at the point location Z_*i*_.

In the case of gentoo penguins, we hypothesize that the hard-core repulsion at small distances is driven by the physical size associated with each individual nest, while at slightly longer length scales territorial behavior increases average inter-nest distance, and at larger scales defense against aerial predators creates positive attraction. The main drivers of nest site selection (i.e. the inhomogeneous intensity of the point process *β*(Z_*i*_)) are likely to be associated with the hydrology of the site, as waterlogging of the nest may prevent important gas exchange over the egg shell [31], and cause hypothermia in chicks that have not yet reached thermal independence [32]. Covariates associated with the underlying suitability of terrain which were selected for inclusion in the statistical model of nesting include elevation, flow accumulation, and a travel cost metric that combines distance to the coast (where penguins haul out of the water after foraging) and slope along their commute back to the nest. While cost-weighted distance and elevation are generally inversely correlated, cost-weighted distance also accounts for those areas in which there is no direct path from the coast to the nest. Flow accumulation uses the aspect of each cell to determine the sum of cells likely to contribute to water-flow into any given cell. The calculation of flow accumulation is dependent on the scale used for analysis; for this reason, flow accumulation was calculated at a variety of scales and model selection used to determine which scale(s) should be retained in the best-fitting model.

Point process models were fitted using the ‘ppm’ function in the ‘statspat’ R package [29]. Appropriate interaction distances are estimated via a model comparison method in which models for all combinations of parameters *h* and *r* are fitted and the model with the lowest Akaike Information Criterion (AIC) score selected. While artificially introduced boundaries in a point pattern can affect model fitting, the point pattern's boundary in this case is created by the coastline of the island and is thus not an artifact of the sampling; correspondingly, no edge correction was used in fitting the models.

## Results

Of the 493 images available for Port Lockroy, the Antarctic penguin breeding site considered for this analysis, 459 were successfully aligned, providing at least 9 views of all areas of the island for generation of the 3D point cloud. The point cloud consisted of 113,338,579 points in 3D space (5,667 points per square meter over an area of 66,395.90 m^2^), which were generalised to a mesh containing 2,495,043 vertices and 4,988,477 faces. This mesh was georectified and converted to a raster with a resolution of ~6 mm.

**Fig 3:**
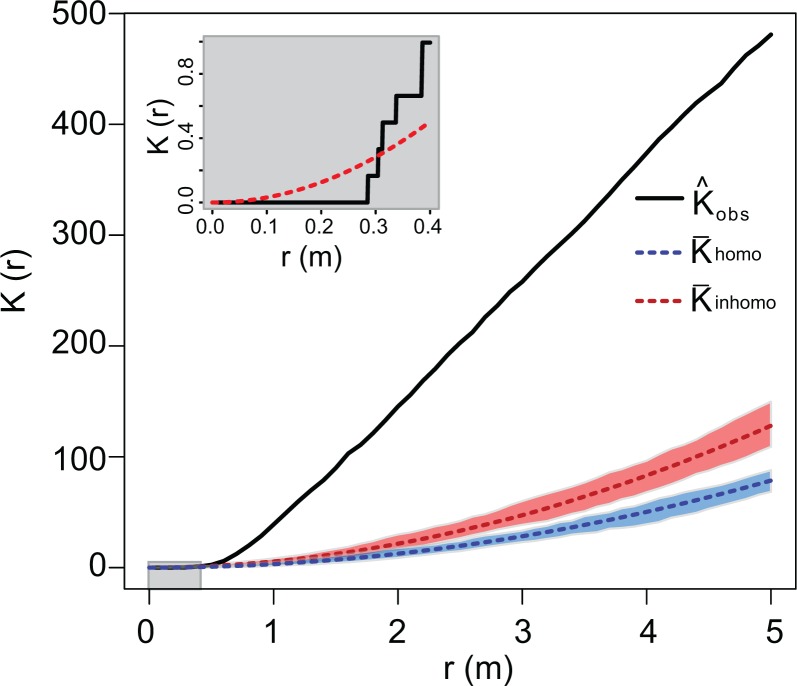
Ripley’s K-function (mean number of points within radius *r* from any point) for the observed point pattern (black), theoretical value under complete spatial randomness (blue), and value under an inhomogeneous Poisson process with no inter-point interaction (red). Confidence intervals generated through 1,000 simulations of point processes. Gentoo nests show over-dispersion (fewer points than expected) at short scales (inset) and under-dispersion (more points than expected) at larger scales.

Ripley’s K indicated significant under-dispersion, or clustering of points, at distances greater than 0.3 m while at smaller distances the points were over-dispersed relative to a stationary Poisson point process (Fig 3). Model selection via AIC indicated that the estimated hard-core distance for the point process model (*h*) was 0.28 m while the Strauss interaction radii (*r*_1_,*r*_2_) were 0.5 m and 1.86 m, respectively.

**Fig 4:**
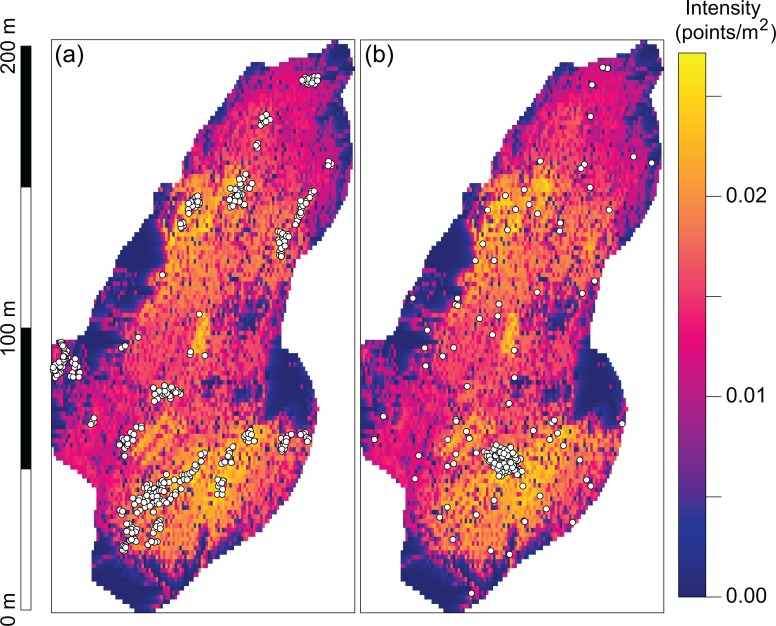
(a) Estimated intensity of inhomogeneous point process of nest locations driven by underlying terrain characteristics. White circles represent the locations of nests in the observed point pattern. (b) A stochastic realization simulated from the fitted Strauss hard-core point process.

Elevation, flow accumulation, and cost distance were found to be statistically significant (p < 0.001) for the occurrence of nests even after allowing for the interaction of points. Flow accumulation was found to be significant at multiple scales (p < 0.001). Nest densities were higher in areas at greater elevations and in those locations unlikely to become water–logged (Fig 4, Table 1). The interaction coefficients (Table 1) are the natural logarithm of the estimated interaction parameters *γ*_1_ and *γ*_2_. Our estimates of $\hat{\gamma_{1}}=-1.13$ (95^th^ percentile CI = [-1.36,-0.88]) and of $\hat{\gamma_{2}}=0.56$ (95^th^ percentile CI = [0.54,0.58]) indicate strong negative interaction at short length scales (≤ 0.5m), and positive interactions at longer scales (0.5 m—1.86 m), leading to a higher density of nesting than would be expected based on first-order inhomogeneity in habitat suitability.

**Table 1:** Parameter estimates for the fitted hybrid Gibbs process model of gentoo nest locations at Port Lockroy, Antarctica. There is zero probability of points existing within 0.28m (h) of each other. From 0.28m to 0.5m (*r*_1_) the probability of occurrence is reduced by $e^{\gamma_{1}}$, and from 0.5m - 1.86m (*r*_2_) the probability of occurrence is increased by $e^{\gamma_{2}}$.

	Coefficient	Lower 95% CI	Upper 95% CI	p Value
Intercept (*μ*)	-4.01	-4.18	-3.84	< 0.001
Elevation (*ρ*_1_)	0.11	0.09	0.13	< 0.001
Flow Accumulation (*ρ*_2_)	-0.00195	-0.00269	-0.00018	< 0.001
Flow Accumulation 4x scale (*ρ*_3_)	-0.0082	-0.0095	-0.0008	< 0.001
Flow Accumulation 16x scale (*ρ*_4_)	-0.0305	-0.0323	-0.0025	< 0.001
Cost Distance (*ρ*_5_)	-0.0012	-0.0014	-0.0010	< 0.001
Interaction (*γ*_1_)	-1.13	-1.36	-0.88	< 0.001
Interaction (*γ*_2_)	0.56	0.54	0.58	< 0.001

## Discussion

**Fig 5:**
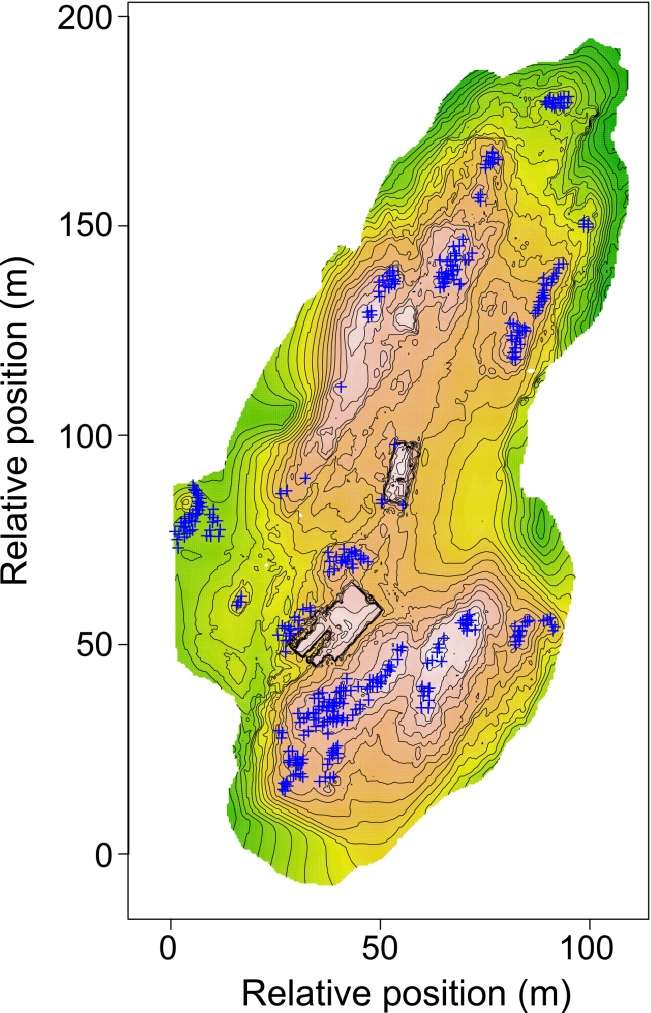
Raster and contour map derived from 3D model produced by Structure-from-Motion.

While the datasets required to study occupancy at large spatial scales are readily available through remote sensing, the environment experienced by individual plants and animals is usually quite localized and may depend on idiosyncratic features not apparent in remotely sensed imagery [16]. Species may occupy sites that at a landscape scale appear unsuitable, but at fine scales contain topographic features that produce microclimates vastly different from the regional average and fall well within physiological requirements. SfM provides a means to understand the interactions between organisms (particularly sessile organisms such as plants or nesting birds) and their environment, and provides data on occupancy and abundance that can be used in a range of spatially-explicit modeling frameworks. While SfM is capable of producing highly detailed 3D models, the standard workflows associated with habitat suitability or point process modeling require data in a planar geometry, causing us to collapse our dataset back to a 2D representation of the environment for analysis (Fig 5). The derived metrics that describe key factors of the microclimate such as hydrology or hillshade may be estimated directly from the high-resolution 3D information collected through SfM. This high resolution topographic information has been shown to improve habitat suitability studies, although previous efforts have utilized LiDAR systems, and have recognized that the associated costs are high. While lower resolution datasets may be constructed through intensive point sampling and interpolation, SfM offers the ability to collect this 3D information of comparable quality to LiDAR at a fraction of the cost.

### Recommendations for best results

To produce high quality 3D models it is important to have sufficient overlap between images (between 60–70%) to allow an adequate number of points to match between images. While camera alignment is theoretically possible with as few as eight matched points, in practice the number of matched points should be orders of magnitude larger. Images should be captured from different locations around the site of interest rather than from an in-situ rotation of the camera. Additionally, an ideal camera should be at least 12 megapixels with a 50 mm film equivalent focal length. Photoscan also supports a fish-eye lens camera model in addition to the standard frame camera model, enabling the use of cameras such as the Go-Pro3 in the SfM pipeline. While results obtained using cameras with a fish-eye lens are of lower quality, the trade-off of lower camera weight may often inform the choice of camera particularly when aerial images are required. The parameters of the camera model, a representation of the transformation of light rays from lens to sensor, are estimated at the same time as the scene structure; however, it is also possible to calibrate cameras in a separate process using a checkerboard pattern image.

The density of reconstructed points is a function of the number of matched points between overlapping images and the distance of the point from the camera. This results in a variable density of points across the reconstructed scene. In fact, there may be portions of the scene that cannot be reconstructed in cases where there is insufficient overlap or coverage in images, occlusion of an area, or insufficient surface texture. While interpolation of the mesh can be used to fill these holes (and can be carried out in Photoscan simultaneously with mesh creation), it is preferable to consider image capture paths prior to surveying to minimize the need for interpolation. In this study, there were several small areas that required interpolation due to the lack of matchable features in snow banks, however these represented a very small proportion of the total surface reconstructed (< 7%) and these areas did not contain penguin nests. It should be noted that the proportion of the surface that was not constructed was far smaller than in most stereo-derived satellite DEMs where cloud cover can result in large holes, particularly in coastal areas.

While it is impossible to calculate errors in the point cloud in the absence of a reference set of measurements of known accuracy, previous studies have compared the accuracies of the SfM approach and traditional LiDAR systems and found the two systems to have similar accuracies [18]. In some instances, greater point densities are achievable through SfM, however it should be noted that errors may not be constant across the scene and may be affected by scene-dependent factors such as the distance of the camera from the scene [18]. Reconstruction may fail completely or result in systematic errors when there is insufficient overlap between images, or when the scene being reconstructed lacks sufficient texture for feature matching. The result may be either a model based on a small subset of the available images with those images that could not be aligned excluded, or a model in which cameras have been incorrectly aligned and erroneous points included. These points can often be manually identified by their position and the projection of the incorrectly aligned camera can be reset. Manual placement of control points between images may then be used to attempt to correct the alignment issues.

### Lessons for seabird ecology

In our demonstration of SfM as applied to gentoo penguin nesting, we find strong evidence of intraspecific interactions that are, in fact, more important to the probability of occupancy than the underlying terrain of the nest site. Simulations from the fitted model show exaggerated clustering, a well-known problem in aggregative point process models [33]. It may be the case that the interactions among individuals are more complex than we have assumed here and that additional repulsive forces would stabilize the point pattern. The smallest inter-nest distance in our dataset was 0.28 m and, accordingly, the hard-core distance in our model was estimated to be 0.28 m. However, the average nearest-neighbor distance is 1 m, consistent with previous estimates that have suggested inter-nest distances of around 1 m [34]. The smallest inter-nest distances found in our dataset could be caused by the reduction from three dimensions to two, with the z component in inter-nest distance lost during the projection onto the planar surface. While the ‘spatstat’ package [30] provides the tools to visualize and summarize point patterns in 3D, the tools for fitting point process models on a 3D surface have not yet been developed.

The interaction effects that we observe may also be due partially to some spatially autocorellated abiotic feature not considered in our model. The use of SfM to produce a virtual representation of the scene allows us to return to the dataset and derive new explanatory metrics that describe additional features of the landscape in a way not be possible with a traditional field survey. In this way, SfM provides an opportunity for reanalysis if new biological hypotheses arise after the survey and initial analysis.

Finally, the realized point process recorded by SfM in the field is likely to be a sub-optimal arrangement of nests that reflects, in part, the residual influence of the initial colonization process. Simulated nesting patterns highlight deviations between the observed point process and the classic Strauss hard-core process and suggest the potential importance of initial conditions, though a more complete analysis of ‘optimal’ nesting strategies and non-equilibrium dynamics is required.

Structure-from-Motion offers a cheap alternative to LiDAR to produce high-resolution 3D information on a landscape [22]). While the software used in this study (Photoscan) is a commercial product, with the use of free software, such as visualSfM, it is now feasible to completely integrate organism and habitat mapping for the price of a suitable camera. Data collected at this resolution are much closer to the scale that is relevant to behavioral choices or dispersal limitations of individual organisms than most datasets currently being used in either habitat suitability or range modeling [16]. The ability to characterize micro-habitat, which can be highly heterogeneous at very small spatial scales, will provide ecologists a much better understanding of the niche requirements of a species.

### Additional ecological applications of SfM

In addition to the production of high-resolution data at scales suitable for individual level habitat suitability modeling, SfM technology could be applied to a range of other ecological problems. SfM has already been demonstrated as a means of extracting morphological information from individual objects such as corals, fossils or skeletons, enabling researchers to record metrics such as the volume of individual regions of an object, as well as providing a means to store and share the 3D structure of an object without the need for access to the original sample (e.g. [15]). This technology also shows potential for rapid, opportunistic abundance surveys for static organisms, such as plants, nesting seabirds, and hauled out seals to name just a few. Traditional panoramic photography has long been used for the census of organisms [35] but complex topography often makes it difficult to align the perspectives of each overlapping image to identify portions of the scene that may have been missed. Through the use of SfM to reconstruct the scene, the uncertainty in overlap between images can be identified and the coverage of the survey estimated. Incomplete surveys can be then be extrapolated as needed.

While we have demonstrated this technology with oblique imagery captured from the ground, results may be further improved, both in terms of consistency of point density and coverage, by using orthogonal imagery captured from an aerial platform, such as a plane, kite, or UAS. With the rapidly decreasing price and increasing usability and autonomy of UASs such as quadrocopters, this technology offers the potential for mapping relatively large areas at high spatial resolution, and producing both DEMs and orthorectified imagery of a location at much higher spatial resolutions than commercially available satellite imagery. All of these technologies will create new opportunities for understanding the fine-scale spatial ecology of organisms.
